# Plasmapherisis in Covid-19 patient under ECMO: A Moroccan case report experience

**DOI:** 10.1016/j.amsu.2022.103250

**Published:** 2022-01-10

**Authors:** Ilyass Laaribi, Safaa Kachmar, Zakaria Bouayed, Hamza Mimouni, Ikram Mekkaoui, Abdelilah El Rhalete, Amine El Mouhib, Houssam Bkiyar, Brahim Housni

**Affiliations:** a Department of Intensive Care Unit, Mohammed VI University Hospital, Oujda, Morocco; b Faculty of Medicine and Pharmacy, Mohammed First University, Oujda, Morocco; c Mohammed First University Oujda, FMP Oujda, LAMCESM, Oujda, Morocco

**Keywords:** Sars-CoV-2, Covid-19, Cytokine storm, ECMO, Plasmapheresis

## Abstract

**Introduction:**

Sars-CoV-2 induces an intense cytokine response called cytokine storm at the origin of acute respiratory distress syndrome, multiple organ dysfunction syndrome and death. In this context, several treatments have been proposed; and plasmapheresis appears as a promising treatment.

**Case presentation:**

We report the case of a 57-year-old patient admitted for Sars-CoV-2 infection, who requiried the use of mechanical ventilation, assistance by veno-venous extracorporeal membrane oxygenation ECMO and treated by plasmapheresis plugged on the ECMO circuit.

**Discussion:**

We discuss the mechanisms responsible for the Sars-CoV-2 induced cytokine storm leading to an acute respiratory distress syndrome and the main therapeutic alternatives with emphasis on plasmapheresis.

**Conclusion:**

Reduction of cytokines by plasmapheresis may be very useful in the management of Covid-19 infection if it is undertaken early even on an ECMO circuit.

## Highlights

• Most patients infected with Sars Cov-2 have a mild to moderate form but some patients develop acute respiratory distress syndrome.
• Cytokine storm is at the origin of this acute respiratory distress syndrome.
• Reduction of cytokines by plasmapheresis may be very useful in the management of COVID-19 infection even on an ECMO circuit.

## 1 Introduction

By the end of November 2019, a new coronavirus appeared in Wuhan, China, which is Sars-CoV-2. This viral infection was rapidly responsible of a global pandemic [1]. Most people infected with this new virus had mild to moderate clinical forms. However, some patients developed an acute respiratory distress syndrome requiring hospitalization in resuscitation department [2]. It is considered that this respiratory aggravation is due to an inflammatory phenomenon commonly known as “cytokine storm” (3). In this context, several treatments have been proposed; and plasmapheresis appears as a promising treatment [4].

We report the case of a 57-year-old patient admitted for Sars-CoV-2 infection, who requiried the use of mechanical ventilation, assistance by veno-venous extracorporeal membrane oxygenation ECMO and treated by plasmapheresis on the ECMO circuit.

## 2 Case presentation

A 57 years old female patient, with type 2 diabetes under metformin 1500mg per day since the age of 49, was admitted to resuscitation department for severe Sars-CoV-2 infection. The patient reported cough, fever and severe asthenia in the previous 7 days and a notion of contact with her daughter tested positive for Sars-CoV-2 10 days earlier. At her admission, the patient was conscious, with fever at 39.1 °C, blood pressure at 138/74mmhg, heart rate at 116 beats per minute, respiratory rate at 41 cycles per minute, arterial oxygen saturation was 65% in ambient air. The patient initially required a non-invasive ventilation NIV (FiO2 = 100%, PEEP = 6, AI = 10).

**Fig. 1:**
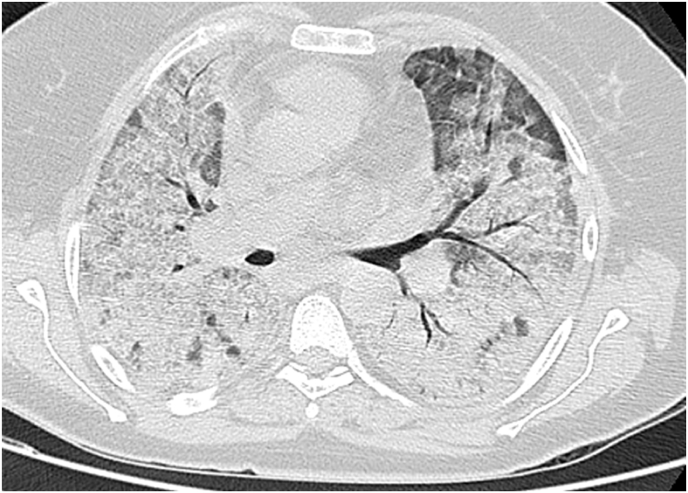
CT image showing involvement of the lungs superior than 75%, classified CORADS 6.

The chest CT showed involvement of the lungs superior than 75%, classified CORADS 6 without signs of pulmonary embolism (Fig. 1).

The arterial blood gases ABG under NIV was PH = 7.39 PaO2 = 68 mmHg PaCO2 = 31 mmHg. The biological parameters found a leukocytosis at 15600/mm³, lymphopenia at 310/mm³, a reactive C protein at 305mg/l, a ferritinemia at 2744 μg/l, a LDH 1268UI/L and a procalcitonin at 15.8ng/ml.

The patient was put under treatment based on antibiotherapy by Ceftriaxon 2g/d and levofloxacin 500mgx2/d, corticotherapy by Dexamethasone 6mg/d, anticoagulants by Enoxaparin 6000UI ×2/d, vitamin C and zinc.

The evolution was marked by severe hypoxemia despite NIV, leading to intubation and conventional protective mechanical ventilation (Vt 360ml, RR at 24 cpm, PEEP at 10, FiO2 at 100%). The ABG after intubation shows a PaO2/Fio2 ratio at 75 with respiratory acidosis, which persisted even after prone position. The need of a femoro-femoral veno-venous ECMO was discussed by the medical staff, with the implantation of 22 F return cannula and 28 F drainage cannula.

**Fig. 2:**
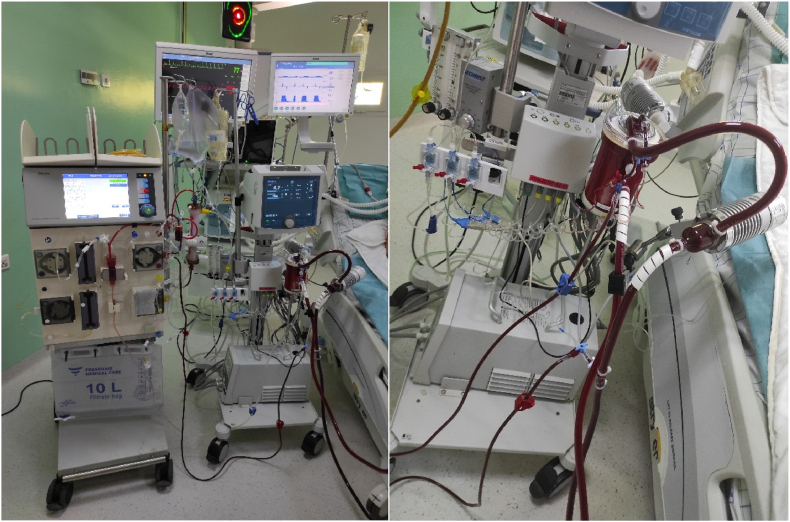
Connection of plasmapheresis device to ECMO circuit.

Plasmapheresis was suggested as therapeutic option to treat the cytokine storm, given the high levels of IL 6 at 438 pg/ml. The plasmapheresis machine was plugged on the ECMO prepump line (Fig. 2), we used a standard plasma exchange kit with a membrane plasma separation filter, the replacement solution consisted of fresh frozen plasma, and the plasma volume was calculated at 40mL/kg of body weight. Each session lasted around 2 hours, the patient benefited from a total of 5 sessions, at a rate of one session per day. A bolus of 1g of methylprednisolone was administered 30 minutes before each session with close blood sugar monitoring.

3 days after the last plasmapheresis session, sedation was stopped. The evolution was favorable with improved lung compliance. The patient was explanted later after a successful ECMO weaning test. She has been tracheotomized because of neumroyopathy and she is evolving favorably at the time of writing this article.

## 3 Discussion

ECMO is an invasive extracorporeal oxygenation technique and is the only remaining alternative for severe hypoxemic patients with acute respiratory distress syndrome ARDS [5]. It was widely used during Covid-19 pandemic. However, the ECMO is not a curative treatment but a support technique. It offers the prospect of prolonging the time of therapy and regeneration of insufficient systems.

Covid-19 infection leads to an excessive inflammatory response with a huge increase of pro-inflammatory cytokines, including IL-6, IL-1, TNF-α and interferon, which generates an influx of various immune cells from circulation to infection site with destructive effects on human tissues, which could explain lung damage and the occurrence of ARDS [6]. This phenomenon, called a cytokine storm, is observed around 7th to 10th day symptom progression, and is considered the main cause of disease severity and death in COVID-19 patients [7]. The early management of cytokine storm by using immunomodulators, cytokine antagonists, leads to reducing mortality in COVID-19 patients [3]. There are currently few studies that demonstrate the benefit of plasmapheresis in the treatment of Covid-19 patients [8].

Plasmapheresis is an extracorporeal purification method that selectively eliminates abnormal substances. It would eliminates inflammatory factors, blocks the process of “cytokine storm” [9] and reduces the damage caused by the inflammatory response. This therapy can be used for severe and critical patients in the early stages of the disease.

In our case, a veno-venous ECMO was implanted quickly before refractory hypoxemia. The choice of plasmapheresis was made because of the availability of material and machines, trained personnel and promising results of this technique in our department [10]. The connection to the ECMO circuit was carried to avoid the implantation of an additional catheter and its complications [11].

## 4 Conclusion

The Covid-19 infection has become a major cause of death in few months. Cytokine storm induced by Sars-CoV2 may lead to high mortality due to the occurrence of ARDS and organ dysfunction. Reduction of cytokines by plasmapheresis may be very useful in the management of Covid-19 infection if it is undertaken early even on an ECMO circuit.

This work is reported in line with the 2020 SCARE guidelines [12].

## Ethical approval

Not applicable, this is a case report.

## Sources of funding

This research was not funded.

## Author contribution

**ILYASS LAARIBI:** study conception, data collection and analysis, writing and editing. **SAFAA KACHMAR:** data collection and writing. **ZAKARIA BOUAYED:** writing. **HAMZA MIMOUNI:** contributor. **MEKKAOUI IKRAM:** contributor. **EL RHALET ABDELILAH:** Contributor. **AMINE EL MOUHIB:** contributor. **HOUSSAM BKIYAR:** Supervision and review data validation. **HOUSNI BRAHIM:** Supervision and review data validation.

## Research registration number

This is not an interventional study. We only reported the patients’ findings from our database as a case report.

## Guarantor

Ilyass Laaribi, Safaa Kachmar.

## Consent

Written informed consent was obtained from the patient for publication of this case report and accompanying images. A copy of the written consent is available for review by the Editor-in-Chief of this journal on request.

## Provenance and peer review

Not commissioned, externally peer reviewed.

## Declaration of competing interest

The authors declare no conflict of interest.
